# A quantitative evaluation of the impact of vaccine roll-out rate and coverage on reducing deaths: insights from the first 2 years of COVID-19 epidemic in Iran

**DOI:** 10.1186/s12916-023-03127-8

**Published:** 2023-11-13

**Authors:** Mahan Ghafari, Sepanta Hosseinpour, Mohammad Saeid Rezaee-Zavareh, Stefan Dascalu, Somayeh Rostamian, Kiarash Aramesh, Kaveh Madani, Shahram Kordasti

**Affiliations:** 1 Big Data Institute and Pandemic Sciences Institute, Nuffield Department of Medicine, University of Oxford, Oxford, UK; 2 Department of Biology, University of Oxford, Oxford, UK; 3 School of Dentistry, The University of Queensland, Herston, QLD 4006 Australia; 4 Middle East Liver Diseases (MELD) Center, Tehran, Iran; 5 Department of Medicine, National Heart and Lung Institute, Imperial College London, London, UK; 6 The James F. Drane Bioethics Institute, PennWest University, Edinboro, PA USA; 7 United Nations University Institute for Water, Environment and Health (UNU-INWEH), Hamilton, ON Canada; 8 Comprehensive Cancer Centre, School of Cancer and Pharmaceutical Sciences, King’s College London, London, UK

**Keywords:** COVID-19, Vaccination, Excess mortality, Counterfactual scenarios, Decision-making

## Abstract

**Background:**

Vaccination has played a pivotal role in reducing the burden of COVID-19. Despite numerous studies highlighting its benefits in reducing the risk of severe disease and death, we still lack a quantitative understanding of how varying vaccination roll-out rates influence COVID-19 mortality.

**Methods:**

We developed a framework for estimating the number of avertable COVID-19 deaths (ACDs) by vaccination in Iran. To achieve this, we compared Iran’s vaccination roll-out rates with those of eight model countries that predominantly used inactivated virus vaccines. We calculated net differences in the number of fully vaccinated individuals under counterfactual scenarios where Iran’s per-capita roll-out rate was replaced with that of the model countries. This, in turn, enabled us to determine age specific ACDs for the Iranian population under counterfactual scenarios where number of COVID-19 deaths are estimated using all-cause mortality data. These estimates covered the period from the start of 2020 to 20 April 2022.

**Results:**

We found that while Iran would have had an approximately similar number of fully vaccinated individuals under counterfactual roll-out rates based on Bangladesh, Nepal, Sri Lanka, and Turkey (~ 65–70%), adopting Turkey’s roll-out rates could have averted 50,000 (95% confidence interval: 38,100–53,500) additional deaths, while following Bangladesh’s rates may have resulted in 52,800 (17,400–189,500) more fatalities in Iran. Surprisingly, mimicking Argentina’s slower roll-out led to only 12,600 (10,400–13,300) fewer deaths, despite a higher counterfactual percentage of fully vaccinated individuals (~ 79%). Emulating Montenegro or Bolivia, with faster per capita roll-out rates and approximately 50% counterfactual full vaccination, could have prevented more deaths in older age groups, especially during the early waves. Finally, replicating Bahrain’s model as an upper-bound benchmark, Iran could have averted 75,300 (56,000–83,000) deaths, primarily in the > 50 age groups.

**Conclusions:**

Our analysis revealed that faster roll-outs were consistently associated with higher numbers of averted deaths, even in scenarios with lower overall coverage. This study offers valuable insights into future decision-making regarding infectious disease epidemic management through vaccination strategies. It accomplishes this by comparing various countries’ relative performance in terms of timing, pace, and vaccination coverage, ultimately contributing to the prevention of COVID-19-related deaths.

**Supplementary Information:**

The online version contains supplementary material available at 10.1186/s12916-023-03127-8.

## Background

On 4 May 2023, the World Health Organization (WHO) determined that the public health emergency phase of the COVID-19 pandemic was over and laid out recommendations for countries on their transition into the long-term management of COVID-19, among other infectious diseases [1]. While the announcement that the healthcare emergency was declared over may have brought some relief to the public, it did not signify an end to the pandemic. As such, governments still need to stay vigilant and support the global effort to take necessary actions for suppressing COVID-19 transmission and reducing the burden of the disease. Among the various public health measures that were implemented throughout the pandemic, maximising the impact of vaccination worldwide has been a key factor in bringing the public health emergency to an end [2]. In this respect, lessons derived from national COVID-19 vaccination campaigns worldwide are crucial as they demonstrate the successes and shortcomings of different countries in reducing the burden of disease during a public health emergency.

Since early 2021, vaccination has been a major contributor to reducing the burden of COVID-19 globally [3, 4]. However, its benefits were not equitably distributed to every country partly due to challenges with the production, distribution, and affordability of vaccines [5, 6] despite efforts to fairly allocate them globally [7] and partly due to vaccine hesitancy [8], which remains a threat to global health. While many studies have focused on quantifying the effectiveness of vaccination campaigns by estimating the number of prevented deaths as a result of vaccination [3, 9–11], fewer studies focused on quantifying the impact of national vaccination programmes’ speed and timings on reducing the burden of COVID-19 [12–15]. These studies mainly investigated the economic and epidemiological impacts of vaccine roll-out timing and speed on disease burden under hypothetical scenarios of fixed or time-varying roll-out rates. However, very limited attention has been given to quantitatively comparing countries’ relative performance based on their vaccine roll-out rates on reducing the burden of COVID-19. Such comparisons would allow for the identification of countries with best practices in implementing effective vaccination programmes, highlight global disparities in vaccination roll-outs, provide a basis for evaluating the impact of specific policies and strategies related to vaccination roll-out speed and timing, and promote a more data-driven decision-making in public health.

By late April 2022, Iran had achieved a vaccination coverage of nearly three quarters of its eligible population against COVID-19, which marked a relatively high level compared to many other upper-middle-income countries (UMICs) and lower-middle-income countries (LMICs) [16]. During this period, approximately 80% of administered doses consisted of the BBIBP-CorV inactivated virus vaccine, while about 9% were home-grown vaccines, primarily the BIV1-COvIran inactivated virus vaccine (Additional file 1: Tables S1 and S2). The remaining doses were mainly composed of AZD1222 and Sputnik V viral vector vaccines. However, despite the high overall vaccination coverage, Iran’s performance fell short when compared to nearly all other LMICs and UMICs with similar vaccination coverage. Having reported nearly 130,000 confirmed deaths and an excess death mortality twice as high by December 2021 [17], Iran ranked 29th out of all 44 UMICs in terms of the number of averted deaths by vaccination per person (Additional file 1: Fig. S1) [3].

While several countries initiated the process of purchasing vaccines towards the end of 2020 and early 2021 through local production, bilateral advance purchase agreements, and the COVID-19 Vaccine Global Access Facility (COVAX) [18, 19], Iran faced several challenges with securing an adequate and timely supply of vaccines. This was partly due to limited global vaccine production capacity and high demand and in part due to geopolitical factors that were unique to Iran [20]. A prime example of geopolitical tensions was the decision to ban the importation of vaccines from the USA and UK to Iran [21]. The country was also not included in the first interim distribution forecast list from COVAX and received its first batch on 5 April 2021 [22] while several other countries received them nearly a month earlier [23, 24]. These factors, along with several others such as delayed home-grown production of vaccines and importations from other countries, contributed to the postponed commencement of the vaccination campaign in Iran. Consequently, 6 months after the start of their vaccination, less than 5% of the Iranian population had been fully vaccinated [20].

A range of public health interventions such as lockdowns, social distancing, contact tracing, and mask wearing in public spaces played a crucial role in mitigating the burden of COVID-19 with varying degrees of effectiveness [25]. As the mass production of vaccines ramped up in 2021, countries began to plan the relaxation of restrictions in tandem with the accelerated pace of vaccination roll-out. During this period, the pivotal role of effective vaccination strategies in reducing the burden of disease became increasingly important. This context underscores the importance of evaluating Iran’s vaccination campaign, as it provides a unique opportunity to understand the impact of delayed vaccination on reducing the number of vaccine-preventable deaths. It can also shed light on the factors that contributed to the increased burden of COVID-19 relative to countries with faster vaccination roll-out and identify potential problems and best practices in vaccination strategies that can be applied more broadly to other countries in the future.

In this work, we developed a framework to retroactively calculate the age-stratified number of avertable deaths in Iran had the country followed the same per capita roll-out rates as other countries. By selecting eight model countries that predominantly vaccinated their populations with inactivated virus vaccines, we examined the impact of vaccination programme start dates, roll-out rates, and overall coverage on avertable COVID-19 deaths (ACDs) in Iran. Our main focus was to estimate the number of avertable deaths by vaccination and not other public health interventions such as lockdowns which have also been shown to play a major role in shaping the burden of disease in Iran [26]. In the end, we discussed a few key public health decisions that may have contributed to the delayed start of vaccination in Iran.

## Methods

We collected all-cause mortality data from Iran’s National Organisation for Civil Registration (https://www.sabteahval.ir/en) and the number of fully vaccinated individuals from Our World in Data [16] and the WHO dashboard [27]. We also obtained the economic status of each country from the World Bank income group in 2020 [28], a year prior to the start of vaccination campaigns in most countries. We selected candidate model countries with comparable income levels and predominant vaccine types to Iran, particularly those that mainly used inactivated virus vaccines such as the BBIBP-CorV vaccine by Sinopharm, as documented in the UNICEF COVID-19 Vaccine Market Dashboard [3, 29]. Since Iran was classed as UMIC before the commencement of vaccine distribution up to 2020 and LMIC from 2021 onwards, we included candidate model countries from both LMICs and UMICs with comparable income levels [28, 30, 31]. These countries include Argentina (UMIC), Bangladesh (LMIC), Bolivia (LMIC), Montenegro (UMIC), Nepal (LMIC), Sri Lanka (LMIC), and Turkey (UMIC). We also included Bahrain, a high-income country (HIC), as an upper-bound benchmark for vaccine coverage and pace relative to Iran.

We assumed that the vaccination roll-out in selected countries would follow the same pattern where the older age groups and those at risk would be vaccinated first in a descending age order [32]. Since the information on the roll-out start dates for Iran is only available for the first dose in each age group, we assumed that the time difference in receiving the full dose (two doses for most vaccines, one or three for a few other manufacturers) between each consecutive age group is the same as the time difference for the primary dose (see Additional file 1: Table S3).

To calculate the net difference in the number of fully vaccinated individuals between Iran and modelled countries over time, we first took the per capita daily number of fully vaccinated individuals in model country *M*, *r*_*M*_(*t*) = *n*_*M*_(*t*)*/P*_*M*_, where *n*(*t*) is the daily number of fully vaccinated people and *P* is the total population size of the country. We then re-normalised the per capita number of fully vaccinated individuals in country *M* with respect to Iran’s population size such that the counterfactual number of fully vaccinated Iranians based on model country *M* would be, *ñ*_*Iran*_(*t*) = *r*_*M*_(*t*) *P*_*Iran*_. The net difference in the daily number of fully vaccinated Iranians in age group *i*, Δ*n*^(*i*)^(*t*), based on the counterfactual vaccine roll-out rate of model country *M* then becomes:

$$\Delta n^{(i)} \left(t\right)=\widetilde{n}^{(i)}_{Iran} \left(t\right)-n^{(i)}_{Iran}\left(t\right)=\widetilde{\alpha}^{(i)} \left(t\right) r_M \left(t\right) P_{Iran}-\alpha^{(i)} \left(t\right) n_{~Iran}\left(t\right)$$

where *ᾶ*^(*i*)^(*t*) and *α*^(*i*)^(*t*) represent the fraction of newly vaccinated individuals under counterfactual and factual scenarios, respectively, and *n*^(*i*)^_*Iran*_ is the number of fully vaccinated individuals in age group *i* in Iran at time *t*. The allocation of newly vaccinated individuals follows a descending age order, starting with the oldest age group receiving the vaccines first. The time for subsequent younger age groups to begin vaccination is determined based on the number of days elapsed since the older age group received the primary dose (Additional file 1: Table S3).

The number of avertable deaths as measured from changes in excess mortality in age group *i* on day *t* as a result of net difference in vaccine roll-out rate under the counterfactual scenario based on model country *M* becomes:

$$D^{(i)}(t)=(1-p^{(i)})\;{\textstyle\sum_{i=1}^{16}}\;\Delta n^{(i)}(t-14)$$

where *p*^(*i*)^ is the vaccine effectiveness against death in age group *i ∈ *{5–9,10–14, …,75–79, 80 +} years old. In other words, if there is no net difference in the number of fully vaccinated individuals on day *t* in age group *i*, there will not be any averted deaths due to vaccination 2 weeks later (i.e. *D*^(*i*)^(*t* + *14*) = 0), and the number of daily deaths at time *t* + 14 will remain the same as the estimated excess deaths in that age group. We assumed that fully vaccinated individuals became protected against deaths from COVID-19 2 weeks after receiving the full dose and that the effectiveness of the vaccine against death is identical across all inactivated virus vaccines and remains the same over time such that *p*^(*i*)^ = 0.923 (95% confidence interval: 0.672–0.982) for *i* > 60 years age groups and *p*^(*i*)^ = 0.801 (95% confidence interval: 0.611–0.898) for *i* ≤ 60 years age groups [33].

We used a previously published model for calculating the number of COVID-19 deaths in Iran using excess mortality data across all age groups eligible to receive vaccination [32]. Age-stratified excess mortality also enabled us to take into account the age-dependent profile of COVID-19 infection fatality ratio (see Figure. 2 in ref [32]). Our analysis covered the period from the start of the pandemic up to 20 April 2022 as the association between excess mortality and reported COVID-19 deaths weakened after the end of the Omicron BA.1/2 wave (see Additional file 1: Fig. S2).

## Results

Our analysis revealed that by 20 April 2022, Iran had 292,666 (95% confidence interval: 262,414–322,919) deaths associated with COVID-19 based on excess mortality estimates and that 67.5% of the population were fully vaccinated. To further investigate the extent to which Iran’s performance could have been enhanced or diminished if alternative vaccination strategies were employed, we examined counterfactual scenarios whereby Iran’s vaccine roll-out rate was replaced with that of the model countries to see if Iran would have had more (or fewer) ACDs.

Our findings showed that for a fixed overall percentage of fully vaccinated individuals, faster roll-out rates were associated with higher ACDs. While the percentage of fully vaccinated Iranians based on a counterfactual vaccine roll-out rate from Bangladesh, Nepal, Sri Lanka, and Turkey would have roughly been the same (~ 65–70%), Iran could have averted as many as 50,000 (95% confidence interval: 38,100–53,500) deaths if it had followed Turkey’s per capita roll-out rates and would have had as many as 52,800 (17,400-189, 500) more deaths if it had followed Bangladesh (Table 1). This corresponds to a nearly 17% reduction in Iran’s excess mortality following Turkey’s and an 18% increase in excess mortality following Bangladesh’s rates. The reason for this is Turkey started their vaccination programme two months earlier than Bangladesh (and Iran) and had a much faster per capita roll-out rate. Following Argentina’s per capita roll-out rates, Iran would have only had a 4% reduction in excess mortality and averted as many as 12,600 (95% CI: 10,400–13,300) deaths despite the fact that Argentina, similar to Turkey, had an early vaccination start date. This is because Argentina had a much slower roll-out rate and, as a result, fewer deaths would have been averted during the Alpha and Delta waves in Iran, particularly in the older age groups (Table 2; also see Additional file 1: Fig. S2 and S3).

**Table 1:** Cumulative and age-stratified avertable COVID-19 deaths^a^ (ACD) in Iran (and 95% confidence interval) based on the per capita vaccine roll-out rates from eight model countries

Model country	Fully vaccinated^b^	ACD < 50 age groups	ACD > 50 age groups	Cumulative ACD
Argentina	78.6%	3200 (2300, 3400)	9200 (8100, 10,100)	12,600 (10,400, 13,300)
Bahrain	86.2%	12,200 (8900, 12,900)	63,200 (47,700, 70,100)	75,300 (56,000, 83,000)
Bangladesh	70.5%	− 20,000 (− 80,700, − 5800)	− 32,800 (− 108,800, − 11,700)	− 52,800 (− 189,500, − 17,400)
Bolivia	50.2%	− 8400 (− 37,800, − 1800)	1800 (− 21,600, 5500)	− 6600 (− 59,400, 3600)
Montenegro	46.9%	− 9100 (− 42,500, − 1800)	33,600 (32,800, 40,700)	31,000 (− 8900, 31,600)
Nepal	66.2%	− 12,400 (− 50,300, − 3600)	7200 (− 10,300, 8900)	− 5200 (− 60,600, 5300)
Sri Lanka	70.0%	2300 (1700, 2500)	21,800 (17,200, 21,900)	24,200 (18,900, 24,400)
Turkey	64.7%	3100 (2100, 3800)	46,200 (35,100, 51,500)	50,000 (38,100, 53,500)

^a^Negative ACD represents counterfactual scenarios in which adopting the vaccination roll-out of a model country leads to a higher number of deaths than what Iran experienced based on its own (factual) roll-out rate

^b^This is based on the counterfactual rescaled vaccination rate of the model countries for the Iranian population by the end of the study period (20 April 2022). The percentage of fully vaccinated individuals in Iran by the end of this period was 67.5%

**Table 2:** Age-stratified avertable COVID-19 deaths^a^ (ACD) in Iran based on the per capita vaccine roll-out rates from model countries at different stages of the epidemic in Iran. This includes ACDs during the Alpha and Delta waves (from 19 February 2021, to 22 December 2021) and the Omicron BA.1 wave (from 5 January 2022, to 20 April 2022)

Country	ACD < 50 age groups during Alpha and Delta	ACD < 50 age groups during Omicron	ACD > 50 age groups during Alpha and Delta	ACD > 50 age groups during Omicron
Argentina	2900 (2100, 3100)	300 (200, 300)	8800 (7800, 9700)	400 (300, 400)
Bahrain	11,600 (8500, 12,400)	500 (400, 500)	62,300 (47,000, 69,000)	900 (700, 1000)
Bangladesh	− 12,200 (− 49,300, − 3500)	− 7100 (− 28,900, − 2100)	− 19,600 (− 71,300, − 5700)	− 12,900 (− 36,500, − 5800)
Bolivia	− 900 (− 7600, 300)	− 7100 (− 28,600, − 2000)	9600 (7900, 11,500)	− 9600 (− 28,900, − 4100)
Montenegro	0 (− 5700, 900)	− 8700 (− 35,300, − 2500)	43,800 (33,500, 47,500)	− 3100 (− 13,600, − 700)
Nepal	− 6400 (− 26,000, − 1800)	− 5400 (− 21,800, − 1600)	9200 (− 1800, 9400)	− 1800 (− 7900, − 400)
Sri Lanka	2300 (1700, 2400)	0 (0, 100)	21,400 (16,900, 21,500)	400 (300, 400)
Turkey	4500 (3300, 4800)	− 700 (− 2700, − 200)	45,900 (34,800, 51,200)	200 (200, 300)

^a^Negative ACD represents counterfactual scenarios in which adopting the vaccination roll-out of a model country leads to a higher number of deaths than what Iran experienced based on its own (factual) roll-out rate

We also found that following either Montenegro’s or Bolivia’s per capita roll-out rates, Iran could have averted many more deaths in the older age groups despite having a 17–20% lower percentage of fully vaccinated individuals (see Table 1). This is because faster roll-out rates based on these model countries would have enabled many more individuals to be protected in the > 50 age groups during the Alpha and Delta waves. However, due to their lower overall percentage of fully vaccinated individuals, as many as 8700 (95% CI: 2000–35,300) more individuals in the < 50 age groups could have lost their lives during the Omicron BA.1 wave (see Table 2). The reason for the wide uncertainty in the number of ACDs for these age groups is that if the vaccine effectiveness against death were very high (up to 90% protective against deaths), then having a lower coverage in younger age groups could have ended with many more deaths during the Omicron BA.1 wave (see the “Methods” section and Table 2).

To further investigate the impact of faster vaccine roll-out rates on ACDs in Iran, we also compared Bolivia’s and Nepal’s counterfactual vaccine roll-out rates. While both countries had similar overall outcomes in terms of cumulative ACDs by mid-April 2022 (see Table 1), following Nepal’s rates, Iran could have had as many as 6400 (1800–26,000) more deaths during Alpha and Delta waves in younger age groups. On the other hand, following Bolivia’s rates, Iran would have had only 900 (300–7600) more deaths over the same period and age groups (see Table 2). This is despite the fact that Bolivia would have had 16% lower overall coverage compared to Nepal under counterfactual scenairos (see Table 1). This is also the same reason why Iran performed much worse than many other LMICs and UMICs with similar or even lower percentages of fully vaccinated individuals because during these two waves, particularly Delta, where the country had its highest per capita death rates (see Additional file 1: Fig. S2), it could have averted many more deaths if it were to vaccinate the population earlier.

Finally, we also compared Iran’s vaccination roll-out against Bahrain. This country had one of the fastest and most effective vaccination campaigns in the Eastern Mediterranean region [16], which can, therefore, provide a reasonable upper bound for the maximum number of ACDs in Iran. We found that following Bahrain’s model, a total of 75,300 (56,000–83,000) deaths could have been averted in Iran during the first 2 years of the pandemic --corresponding to approximately 26% reduction in Iran’s excess mortality (Table 1). The majority of these ACDs would have affected the > 50 age groups during the Alpha and Delta waves from mid-February to late December 2021 (Table 2; see also Additional file 1: Fig. S3).

## Discussion

In this study, we developed a quantitative framework to retrospectively estimate the number of COVID-19 vaccine-preventable deaths in Iran by using per-capita vaccine roll-out rates from other countries as a basis for comparison. Iran was selected as a case study due to the delayed start of its national vaccination campaign which has been suggested to have contributed to a higher mortality and disease burden [20, 32, 34]. Our framework provides a simple and rapid assessment to gauge the impact of relative vaccination coverage, pace, and timing from different countries on avertable deaths. The method provides insight into the identification of countries with effective vaccination programmes, a basis for evaluating the impact of specific policies related to vaccination roll-out speed and timing, and encourages transparency and accountability in the management of vaccination programmes. The applicability of our method extends beyond the COVID-19 pandemic and can also aid future decisions around vaccination strategies aimed at reducing the burden of other infectious diseases with epidemic and/or pandemic potential such as polio, influenza, and measles where vaccination timing and coverage among targeted age-groups is important [35–37].

Our findings revealed that had Iran followed the per capita roll-out rates of certain model countries, it could have potentially averted a significantly higher number of deaths, particularly during the first 9 months of 2021, due to their faster COVID-19 vaccine roll-out rates. The impact of faster roll-outs would have been far greater in averting deaths in the older age groups due to their elevated infection fatality rate [38, 39], whereas achieving higher overall coverage would have been more effective in preventing deaths in younger age groups. For instance, our results indicated that Iran could have saved nearly 31,000 more lives if it had followed the fast roll-out rates of Montenegro despite having a 20% lower overall coverage. This is because many more lives could have been saved in the older age groups during the Alpha and Delta waves despite having more deaths in the younger age groups during the Omicron BA.1 wave in Iran.

Comparisons with Bahrain, a country with a highly effective vaccination campaign, demonstrated that nearly 75,000 more deaths could have been averted in Iran by following a similar model. These findings showed the potential benefits of early vaccination start dates but more importantly faster roll-out rates in reducing the disease burden. Another example of an early and effective vaccination campaign is Israel which had a swift vaccination roll-out using predominantly mRNA vaccines. It experienced markedly lower per capita mortality rates during the Delta and Omicron waves compared to many other countries with slower vaccination rates [40]. These observations also align with the findings from observational studies showing that highly vaccinated communities experience significantly fewer deaths [41].

A key strength of the study lied in our reliance on excess mortality estimates rather than reported deaths for calculating ACDs, a method that has been shown to reliably capture the true death toll from COVID-19 in Iran as it avoids under-reporting biases [17, 32, 42]. Our estimates for ACDs should be interpreted as a conservative lower bound, as we did not incorporate the indirect effects of vaccination on reducing transmission rates and the complex interplay of other non-pharmaceutical interventions on lowering virus circulation which can in turn further contribute to increasing ACDs. To address these limitations, a comprehensive transmission dynamic model would be necessary, demanding rich data on parameters such as population infection history, waning immunity, and vaccine effectiveness across variants and vaccine types, a wealth of information lacking for many resource-constrained regions, including Iran [10]. Therefore, given the dearth of empirical data for precise parameter estimations [42] and risk of model over-parametrisation, we believe our model provides a fast and simple framework for estimating ACDs for Iran and other countries with similar limitations using excess mortality data which is readily available for most countries around the world and does not greatly suffer from under-reporting biases [43, 44].

One of the major contributors to the delayed vaccination programme in Iran was the prioritisation of domestic vaccine production over securing early deals with other vaccine producers through COVAX and bilateral agreements [45], particularly the emphasis on the local production of BIV1-COvIran vaccine from Barekat Pharmaceutical, a home-grown vaccine company which faced scaling challenges as well as concerns over transparency and data sharing [46, 47]. Among the other missed opportunities in Iran’s vaccination programme in 2021 was the banning of 150,000 doses of the Pfizer-BioNTech vaccine donated through Iran’s Red Crescent organisation which could have prevented thousands of COVID-19 deaths [20]. Given that mRNA vaccines elicit stronger humoral immunity relative to inactivated virus vaccines [50–52], it is plausible that they would have prevented more infections and deaths if they were more widely used or in combination with inactivated virus vaccines as heterologous vaccine regimens [53]. Another example of such missed opportunities was the decision not to participate in phase 3 clinical trials of inactivated virus vaccines. Iran’s Ministry of Health had a policy to only participate in such trials only if the manufacturer made a commitment to jointly collaborate with Iran in the technology transfer and production of vaccines. Such an agreement was only reached with the Finley Institute of Cuba which ran its phase 3 trials in Iran [48, 49]. However, this vaccine was not granted emergency use authorisation until much later and without any large importations from Cuba or local production in Iran, making up only 1.7% of all administered vaccine doses in Iran (see Supplementary Table 2) [20].

In contrast, some of the model countries included in this study such as Argentina, Bahrain, and Turkey all participated in trials for inactivated virus vaccines and benefited from securing more doses at earlier dates compared to Iran. This highlights the advantages of participating in such clinical trials for faster vaccine rollouts. Bangladesh, on the other hand, did not participate in any phase 3 trials [54] and Nepal’s vaccination roll-out was in part affected by the halting of vaccine exports from India which the country relied on in 2021 [55]. Additionally, Montenegro’s swift vaccine roll-out, despite not participating in clinical trials, underscores the importance of rapid deployment of vaccines, especially in smaller-sized nations [56].

Closer examination of Iran’s COVID-19 vaccination experience also provides valuable insights for future public health policy decisions. The delay in initiating the vaccination campaign resulted from preventable factors, including the prohibition of vaccine imports from certain countries despite their proven safety and efficacy, delayed efforts to secure vaccine doses, and an over-reliance on the punctual delivery of domestically developed vaccines. These factors contributed to a lower number of vaccine-preventable deaths in Iran, but their individual impact is not quantified in this study. The causes of delay highlight undue political interference in the health sector, the absence of independent bodies to challenge politically motivated decisions, and a lack of transparency to address corruption allegations. The study underscored the importance of implementing the consensual and culturally sensitive principle of ‘Independent Review’ through the establishment of independent research committees, institutional review boards, and academic organisations, serving as key lessons in research ethics and public health ethics [57].

## Conclusions

In conclusion, this study provided a quantitative framework to compare the performance of different countries based on their timing, pace, and coverage of vaccination and their impact on avertable COVID-19 deaths. It demonstrated the critical role of faster roll-out rates on further reducing COVID-19 deaths, particularly during the waves of infection with the Alpha and Delta variants in older age-groups with highest risk of severe disease outcome and deaths and provided a means to find effective vaccination strategies in managing infectious disease epidemics based on country-level comparisons. More broadly, our modelling framework can provide a tool for policymakers and public health officials to assess the successes and challenges of other countries, address disparities in vaccination, evaluate policy effectiveness, make data-driven decisions, and prepare for future health emergencies.

### Supplementary Information

**Additional file 1: Table S1. **A timeline of vaccine imports to Iran from 3 February 2021 to 16 November 2021. **Table S2.** Total number of administered doses of COVID-19 vaccines in Iran per vaccine name by 29 May 2022 [58]. **Table S3.** Iran’s vaccine roll-out dates per age-group [32]. **Figure S1.** Deaths averted by vaccination for upper and lower middle income countries, including Iran, up to 8 December 2021. Data on deaths averted per person and per vaccine are downloaded from [3]. The economic status of each country is based on the World Bank income groups in 2020 [28]. **Figure S2.** Association between reported cases, deaths, and excess deaths over time and with respect to variants of SARS-CoV-2. (A) Reported cases, deaths, and excess deaths (as quantified by the sum of excess deaths per age group) as percentage of highest peak (Delta) in Iran over time. (B) Proportion of the total number of SARS-CoV-2 variants over time in Iran. Data obtained from [59]. **Figure S3.** Avertable COVID-19 deaths in Iran over time based on the per capita vaccine roll-out rates from model countries. Top panel shows Iran’s weekly excess deaths (black) and counterfactual excess deaths (magenta) had it followed the vaccination rate for each model country. Shaded areas show the 95% confidence interval for the counterfactual excess deaths based on varying degree of vaccine effectiveness against deaths. Central panel shows the avertable deaths based on vaccination rates from a given model country. It shows the difference between weekly excess deaths and counterfactual excess deaths in the top panel. Bottom panel shows the percentage of excess vaccination for a given model country relative to Iran’s vaccination rates per age group. Shaded areas in green (red) show periods where there would have been more (less) vaccination had Iran followed the vaccination rates as the model country.

## Data Availability

All software code and analysis scripts are available in Mathematica version 11 on GitHub (https://github.com/mg878/Vaccine_preventable_Deaths). All-cause mortality data is available on Iran’s National Organisation for Civil Registration (https://www.sabteahval.ir). Number of fully vaccinated individuals are available from Our World in Data (https://ourworldindata.org/coronavirus).

## References

[1] Statement on the fifteenth meeting of the IHR (2005) Emergency Committee on the COVID-19 pandemic. https://www.who.int/news/item/05-05-2023-statement-on-the-fifteenth-meeting-of-the-international-health-regulations-(2005)-emergency-committee-regarding-the-coronavirus-disease-(covid-19)-pandemic. Accessed 28 May 2023.

[2] Lazarus JV, Romero D, Kopka CJ, Karim SA, Abu-Raddad LJ, Almeida G (2022). A multinational Delphi consensus to end the COVID-19 public health threat. Nature.

[3] Watson OJ, Barnsley G, Toor J, Hogan AB, Winskill P, Ghani AC (2022). Global impact of the first year of COVID-19 vaccination: a mathematical modelling study. Lancet Infect Dis.

[4] Notarte KI, Catahay JA, Velasco JV, Pastrana A, Ver AT, Pangilinan FC (2022). Impact of COVID-19 vaccination on the risk of developing long-COVID and on existing long-COVID symptoms: A systematic review. EClinicalMedicine.

[5] Wouters OJ, Shadlen KC, Salcher-Konrad M, Pollard AJ, Larson HJ, Teerawattananon Y (2021). Challenges in ensuring global access to COVID-19 vaccines: production, affordability, allocation, and deployment. Lancet.

[6] Bollyky TJ, Gostin LO, Hamburg MA (2020). The Equitable distribution of COVID-19 therapeutics and vaccines. JAMA.

[7] Fair allocation mechanism for COVID-19 vaccines through the COVAX Facility. https://www.who.int/publications/m/item/fair-allocation-mechanism-for-covid-19-vaccines-through-the-covax-facility. Accessed 28 May 2023.

[8] Wiysonge CS, Ndwandwe D, Ryan J, Jaca A, Batouré O, Anya B-PM (2022). Vaccine hesitancy in the era of COVID-19: could lessons from the past help in divining the future?. Hum Vaccin Immunother.

[9] Vilches TN, Moghadas SM, Sah P, Fitzpatrick MC, Shoukat A, Pandey A (2022). Estimating COVID-19 Infections, hospitalizations, and deaths following the US Vaccination campaigns during the pandemic. JAMA Netw Open.

[10] Jia KM, Hanage WP, Lipsitch M, Johnson AG, Amin AB, Ali AR, et al. Estimated preventable COVID-19-associated deaths due to non-vaccination in the United States. Eur J Epidemiol. 2023;24:1–4. 10.1007/s10654-023-01006-3 PMC10123459 37093505

[11] Meslé MM, Brown J, Mook P, Hagan J, Pastore R, Bundle N, et al. Estimated number of deaths directly averted in people 60 years and older as a result of COVID-19 vaccination in the WHO European Region, December 2020 to November 2021. Euro Surveill. 2021;26:2101021 10.2807/1560-7917.ES.2021.26.47.2101021 PMC8619871 34823641

[12] Liu Y, Procter SR, Pearson CAB, Montero AM, Torres-Rueda S, Asfaw E (2023). Assessing the impacts of COVID-19 vaccination programme’s timing and speed on health benefits, cost-effectiveness, and relative affordability in 27 African countries. BMC Med.

[13] Sah P, Vilches TN, Moghadas SM, Fitzpatrick MC, Singer BH, Hotez PJ (2021). Accelerated vaccine rollout is imperative to mitigate highly transmissible COVID-19 variants. EClinicalMedicine.

[14] Bauer S, Contreras S, Dehning J, Linden M, Iftekhar E, Mohr SB (2021). Relaxing restrictions at the pace of vaccination increases freedom and guards against further COVID-19 waves. PLoS Comput Biol.

[15] Ferreira LS, Darcie Marquitti FM, Paixão da Silva RL, Borges ME, Ferreira da Costa Gomes M, Cruz OG, et al. Estimating the impact of implementation and timing of the COVID-19 vaccination programme in Brazil: a counterfactual analysis. Lancet Reg Health Am. 2023;17:100397. 10.1016/j.lana.2022.100397 PMC9676113 36439909

[16] Mathieu E, Ritchie H, Rodés-Guirao L, Appel C, Giattino C, Hasell J, et al. Coronavirus pandemic (COVID-19). Our World in Data. 2020. Published online at OurWorldInData.org. Retrieved from: https://ourworldindata.org/coronavirus.

[17] Ghafari M, Kadivar A, Katzourakis A (2021). Excess deaths associated with the Iranian COVID-19 epidemic: a province-level analysis. Int J Infect Dis.

[18] Callaway E (2020). The unequal scramble for coronavirus vaccines - by the numbers. Nature.

[19] Dyer O (2021). COVID-19: Countries are learning what others paid for vaccines. BMJ.

[20] Ghafari M, Rezaee-Zavareh MS, Dascalu S, Katzourakis A. Iran’s COVID-19 vaccination programme: using transparency to build public trust in immunisation. The BMJ. 2021. https://blogs.bmj.com/bmj/2021/08/03/irans-covid-19-vaccination-programme-using-transparency-to-build-public-trust-in-immunisation/. Accessed 28 May 2023.

[21] Iran bans import of UK and US COVID-19 vaccines, saying they’re “completely untrustworthy.” France 24. 2021. https://www.france24.com/en/middle-east/20210109-iran-bans-import-of-uk-and-us-covid-19-vaccines-saying-they-re-completely-untrustworthy. Accessed 28 May 2023.

[22] Iran receives first delivery of COVID-19 vaccines through COVAX facility. https://www.unicef.org/iran/en/press-releases/iran-receives-first-delivery-covid-19-vaccines-through-covax-facility. Accessed 31 May 2023.

[23] The first shipment of COVAX vaccines. https://www.unicef.org/supply/stories/first-shipment-covax-vaccines. Accessed 31 May 2023.

[24] First COVID-19 vaccines arrive in Malawi thanks to COVAX Facility. https://www.eeas.europa.eu/eeas/first-covid-19-vaccines-arrive-malawi-thanks-covax-facility_en. Accessed 31 May 2023.

[25] Royal Society Expert Working Group. COVID-19: examining the effectiveness of non-pharmaceutical interventions. 2023. 10.1098/rsta.2023.0211 PMC10446904 37611626

[26] Ghafari M, Hejazi B, Karshenas A, Dascalu S, Kadvidar A, Khosravi MA (2021). Lessons for preparedness and reasons for concern from the early COVID-19 epidemic in Iran. Epidemics.

[27] WHO Coronavirus (COVID-19) dashboard. https://covid19.who.int/. Accessed 31 May 2023.

[28] United Nations: Department of Economic and Social Affairs. World economic situation and prospects (2020). New York.

[29] COVID-19 market dashboard. https://www.unicef.org/supply/covid-19-market-dashboard. Accessed 31 May 2023.

[30] United Nations: Department of Economic and Social Affairs. World economic situation and prospects (2021). New York.

[31] United Nations: Department of Economic and Social Affairs. World economic situation and prospects (2022). New York.

[32] Ghafari M, Watson OJ, Karlinsky A, Ferretti L, Katzourakis A (2022). A framework for reconstructing SARS-CoV-2 transmission dynamics using excess mortality data. Nat Commun.

[33] Al Kaabi N, Oulhaj A, Ganesan S, Al Hosani FI, Najim O, Ibrahim H (2022). Effectiveness of BBIBP-CorV vaccine against severe outcomes of COVID-19 in Abu Dhabi. United Arab Emirates Nat Commun.

[34] Safavi-Naini SAA, Pourhoseingholi MA (2022). The early impact of COVID-19 vaccination on deaths among elderly people in Iran. Gastroenterol Hepatol Bed Bench.

[35] Baptista Risi J, Jr. The control of poliomyelitis in Brazil. Rev Infect Dis. 1984;6(Suppl 2):S400–3. 10.1093/clinids/6.supplement_2.s400 6740081

[36] Ferguson NM, Cummings DAT, Fraser C, Cajka JC, Cooley PC, Burke DS (2006). Strategies for mitigating an influenza pandemic. Nature.

[37] Noh J-W, Kim Y-M, Akram N, Yoo KB, Cheon J, Lee LJ (2019). Determinants of timeliness in early childhood vaccination among mothers with vaccination cards in Sindh province, Pakistan: a secondary analysis of cross-sectional survey data. BMJ Open.

[38] COVID-19 Forecasting Team (2022). Variation in the COVID-19 infection-fatality ratio by age, time, and geography during the pre-vaccine era: a systematic analysis. Lancet.

[39] O’Driscoll M, Dos Santos GR, Wang L, Cummings DAT, Azman AS, Paireau J (2020). Age-specific mortality and immunity patterns of SARS-CoV-2. Nature.

[40] Sandmann FG, Jit M (2022). Rapid COVID-19 vaccine rollout: immense success but challenges ahead. Lancet Infect Dis.

[41] Suthar AB, Wang J, Seffren V, Wiegand RE, Griffing S, Zell E (2022). Public health impact of COVID-19 vaccines in the US: observational study. BMJ.

[42] Ghafari M, Kadivar A, Katzourakis A. Estimates of anti-SARS-CoV-2 antibody seroprevalence in Iran. The Lancet Infectious Diseases. 2021;21:602–3 10.1016/S1473-3099(21)00053-0 PMC7906708 33600759

[43] Msemburi W, Karlinsky A, Knutson V, Aleshin-Guendel S, Chatterji S, Wakefield J (2023). The WHO estimates of excess mortality associated with the COVID-19 pandemic. Nature.

[44] Karlinsky A, Kobak D. Tracking excess mortality across countries during the COVID-19 pandemic with the World Mortality Dataset. Elife. 2021;10:e69336. 10.7554/eLife.69336 PMC8331176 34190045

[45] Torbati Y. Amid covid surge, Iran cut corners to approve yet-unproven vaccine. The Washington Post. 2022. https://www.washingtonpost.com/world/2022/08/20/iran-covid-vaccine-approval/.

[46] Rovetta A, Garavaglia R, Vitale A, Meccia E, Tesfaye BT, Mezzana P, et al. An improved peer-review system to compensate for scientific misconduct in health-sensitive topics. 2022;44:1605601. 10.3389/phrs.2023.1605601 PMC10272364 37332396

[47] Negotiating with major vaccine manufacturers in the world [Persian]. https://dolat.ir/detail/344397. Accessed 31 May 2023.

[48] Mok CKP, Cohen CA, Cheng SMS, Chen C, Kwok K-O, Yiu K (2022). Comparison of the immunogenicity of BNT162b2 and CoronaVac COVID-19 vaccines in Hong Kong. Respirology.

[49] Premikha M, Chiew CJ, Wei WE, Leo YS, Ong B, Lye DC (2022). Comparative effectiveness of mRNA and inactivated whole-virus vaccines against Coronavirus disease 2019 infection and severe disease in Singapore. Clin Infect Dis.

[50] Tan CY, Chiew CJ, Lee VJ, Ong B, Lye DC, Tan KB (2022). Comparative effectiveness of 3 or 4 doses of mRNA and inactivated whole-virus vaccines against COVID-19 infection, hospitalization and severe outcomes among elderly in Singapore. Lancet Reg Health West Pac.

[51] Au WY, Cheung PP-H (2022). Effectiveness of heterologous and homologous COVID-19 vaccine regimens: living systematic review with network meta-analysis. BMJ.

[52] Mostafavi E, Eybpoosh S, Karamouzian M, Khalili M, Haji-Maghsoudi S, Salehi-Vaziri M (2023). Efficacy and safety of a protein-based SARS-CoV-2 vaccine: a randomized clinical trial. JAMA Netw Open.

[53] Iranian Registry of Clinical Trials (IRCT). https://www.irct.ir/trial/54833. Accessed 29 May 2023.

[54] Islam MR, Hasan M, Nasreen W, Tushar MI, Bhuiyan MA (2021). The COVID-19 vaccination experience in Bangladesh: Findings from a cross-sectional study. Int J Immunopathol Pharmacol.

[55] Kansakar S, Dumre SP, Raut A, Huy NT (2021). From lockdown to vaccines: challenges and response in Nepal during the COVID-19 pandemic. Lancet Respir Med.

[56] Calleja N, Gualtieri A, Terzic N, Scoutellas V, Calleja-Agius J (2022). Managing COVID-19 in four small countries: initial response to the pandemic in San Marino, Montenegro. Malta and Cyprus Health Policy.

[57] Universal Declaration on bioethics and Human Rights. 2005. https://www.unesco.org/en/legal-affairs/universal-declaration-bioethics-and-human-rights?hub=66535. Accessed 10 Jun 2023.

[58] Covid-19 Update: Islamic Republic of Iran (No. 832 / 31 May 2022). World Health Organization - Regional Office for the Eastern Mediterranean. http://www.emro.who.int/iran.

[59] Hodcroft E. CoVariants: SARS-CoV-2 mutations and variants of interest. https://covariants.org/. Accessed 18 Jan 2023.

