# Supplementary material for: A quantitative evaluation of the impact of vaccine roll-out rate and coverage on reducing deaths: insights from the first 2 years of COVID-19 epidemic in Iran
Source: BMC Med. 2023 Nov 13;21:429. doi: 10.1186/s12916-023-03127-8 (PMC10642021; doi:10.1186/s12916-023-03127-8)
Supplement: Supplementary file 1 — Additional file 1: Table S1. A timeline of vaccine imports to Iran from 3 February 2021 to 16 November 2021. Table S2. Total number of administered doses of COVID-19 vaccines in Iran per vaccine name by 29 May 2022 [58]. Table S3. Iran’s vaccine roll-out dates per age-group [32]. Figure S1. Deaths averted by vaccination for upper and lower middle income countries, including Iran, up to 8 December 2021. Data on deaths averted per person and per vaccine are downloaded from [3]. The economic status of each country is based on the World Bank income groups in 2020 [28]. Figure S2. Association between reported cases, deaths, and excess deaths over time and with respect to variants of SARS-CoV-2. (A) Reported cases, deaths, and excess deaths (as quantified by the sum of excess deaths per age group) as percentage of highest peak (Delta) in Iran over time. (B) Proportion of the total number of SARS-CoV-2 variants over time in Iran. Data obtained from [59]. Figure S3. Avertable COVID-19 deaths in Iran over time based on the per capita vaccine roll-out rates from model countries. Top panel shows Iran’s weekly excess deaths (black) and counterfactual excess deaths (magenta) had it followed the vaccination rate for each model country. Shaded areas show the 95% confidence interval for the counterfactual excess deaths based on varying degree of vaccine effectiveness against deaths. Central panel shows the avertable deaths based on vaccination rates from a given model country. It shows the difference between weekly excess deaths and counterfactual excess deaths in the top panel. Bottom panel shows the percentage of excess vaccination for a given model country relative to Iran’s vaccination rates per age group. Shaded areas in green (red) show periods where there would have been more (less) vaccination had Iran followed the vaccination rates as the model country. [file 12916_2023_3127_MOESM1_ESM.docx]

**Additional file 1**

**A quantitative evaluation of the impact of vaccine roll-out rate and coverage on reducing deaths: Insights from the first two years of COVID-19 epidemic in Iran**

Mahan Ghafari^1,2,*^, Sepanta Hosseinpour^3^,

^1^Big Data Institute, Nuffield Department of Medicine, University of Oxford, Oxford, UK

^2^Department of Biology, University of Oxford, Oxford, UK

^3^School of Dentistry, The University of Queensland, Herston QLD 4006, Australia

^4^Middle East Liver Diseases (MELD) Center, Tehran, Iran

^5^ National Heart and Lung Institute, Department of Medicine, Imperial College London, London, UK

^6^The James F. Drane Bioethics Institute, PennWest University, Edinboro PA, USA

^7^United Nations University Institute for Water, Environment and Health (UNU-INWEH), Hamilton, Ontario, Canada

^8^Comprehensive Cancer Centre, School of Cancer and Pharmaceutical Sciences, King’s College London, London, UK

*Corresponding author: [mahan.ghafari@ndm.ox.ac.uk](mailto:mahan.ghafari@ndm.ox.ac.uk); [shahram.kordasti@kcl.ac.uk](mailto:shahram.kordasti@kcl.ac.uk)

**Supplementary Tables**

**Table S1: A timeline of vaccine imports to Iran from 02/02/2021 to 16/11/2021.**

| **Item No.** | **Vaccine type - originating country - cargo #** | **Date of entry** | **Quantity** |
| --- | --- | --- | --- |
| 1 | Sputnik V - Russia - 1 | 03/02/2021 | 20,000 |
| 2 | Sputnik V - Russia - 2 | 13/02/2021 | 100,000 |
| 3 | Sinopharm - China - 1 | 28/02/2021 | 250,000 |
| 4 | Sputnik V - Russia - 2 | 04/03/2021 | 200,000 |
| 5 | Bharat - India - 1 | 10/03/2021 | 125,000 |
| 6 | Sputnik V - Russia - 4 | 25/03/2021 | 100,000 |
| 7 | Sputnik V - Russia - 5 | 01/04/2021 | 100,000 |
| 8 | AstraZeneca - South Korea - 1 | 05/04/2021 | 700,800 |
| 9 | Sinopharm - China - 2 | 15/04/2021 | 400,000 |
| 10 | Sputnik V - Russia - 6 | 22/04/2021 | 100,000 |
| 11 | Sputnik V - Russia - 7 | 29/04/2021 | 100,000 |
| 12 | Sinopharm - China - 3 | 02/05/2021 | 1,000,000 |
| 13 | Sinopharm - China - 4 | 16/05/2021 | 1,000,000 |
| 14 | AstraZeneca - Italy - 1 | 16/05/2021 | 1,452,000 |
| 15 | Sputnik V - Russia - 8 | 03/06/2021 | 100,000 |
| 16 | Sputnik V - Russia - 9 | 10/06/2021 | 100,000 |
| 17 | Sinopharm - China - 5 | 17/06/2021 | 1,000,000 |
| 18 | Sinopharm - China - 6 | 24/06/2021 | 1,000,000 |
| 19 | Sinopharm - China - 7 | 08/07/2021 | 1,056,000 |
| 20 | Sinopharm - China - 8 | 10/07/2021 | 500,000 |
| 21 | Sinopharm - China - 9 | 11/07/2021 | 444,000 |
| 22 | Sinopharm - China - 10 | 15/07/2021 | 1,142,800 |
| 23 | Sinopharm - China - 11 | 18/07/2021 | 1,130,600 |
| 24 | Sinopharm - China - 12 | 22/07/2021 | 1,130,400 |
| 25 | AstraZeneca - Japan - 1 | 23/07/2021 | 1,087,570 |
| 26 | Sinopharm - China - 13 | 25/07/2021 | 1,095,200 |
| 27 | AstraZeneca - Russia (private sector) - 1 | 28/07/2021 | 315,000 |
| 28 | Sputnik V - Russia - 10 | 29/07/2021 | 100,000 |
| 29 | AstraZeneca - Japan - 2 | 30/07/2021 | 1,098,300 |
| 30 | AstraZeneca - Japan - 3 | 30/07/2021 | 725,940 |
| 31 | Sinopharm - China - 14 | 01/08/2021 | 1,120,000 |
| 32 | Sinopharm - China - 15 | 05/08/2021 | 1,110,000 |
| 33 | Sinopharm - China - 16 | 08/08/2021 | 1,110,000 |
| 34 | Sinopharm - China - 17 | 12/08/2021 | 1,110,000 |
| 35 | Sinopharm - China - 18 | 12/08/2021 | 500,000 |
| 36 | Sinopharm - China - 19 | 15/08/2021 | 1,110,000 |
| 37 | Sinopharm - China - 20 | 17/08/2021 | 700,000 |
| 38 | Sinopharm - China - 21 | 19/08/2021 | 1,110,000 |
| 39 | Sputnik V - Serbia - 11 | 20/08/2021 | 25,000 |
| 40 | Sinopharm - China - 22 | 22/08/2021 | 1,110,000 |
| 41 | Sinopharm - China - 23 | 26/08/2021 | 2,220,000 |
| 42 | AstraZeneca - China - 1 | 02/09/2021 | 1,449,600 |
| 43 | Sinopharm - China - 24 | 02/09/2021 | 179,596 |
| 44 | Sinopharm - China - 25 | 03/09/2021 | 4,115,088 |
| 45 | AstraZeneca - Russia (private sector) - 2 | 04/09/2021 | 648,000 |
| 46 | Sinopharm - China - 26 | 05/09/2021 | 5,000,000 |
| 47 | Sinopharm - China - 27 | 07/09/2021 | 897,014 |
| 48 | AstraZeneca - Austria - 1 | 08/09/2021 | 500,000 |
| 49 | Sinopharm - China - 28 | 09/09/2021 | 5,000,000 |
| 50 | Sinopharm - China - 29 | 11/09/2021 | 3,196,800 |
| 51 | Sinopharm - China - 30 | 16/09/2021 | 5,000,000 |
| 52 | Sputnik V - Russia - 12 | 16/09/2025 | 300,000 |
| 53 | AstraZeneca - Austria - 2 | 16/09/2021 | 150,000 |
| 54 | Sinopharm - China - 31 | 17/09/2021 | 3,000,000 |
| 55 | Sinopharm - China - 32 | 21/09/2021 | 2,000,000 |
| 56 | Sinopharm - China - 33 | 23/09/2021 | 5,000,000 |
| 57 | Sinopharm - China - 34 | 26/09/2021 | 6,000,000 |
| 58 | Sputnik V - Serbia - 13 | 27/09/2021 | 25,000 |
| 59 | Sinopharm - China - 35 | 28/09/2021 | 2,000,000 |
| 60 | Sinopharm - China - 36 | 30/09/2021 | 6,000,000 |
| 61 | AstraZeneca - Greece - 1 | 01/10/2021 | 150,000 |
| 62 | AstraZeneca - Austria - 3 | 03/10/2021 | 350,000 |
| 63 | Bharat - India - 2 | 09/10/2021 | 1,000,000 |
| 64 | Sinopharm - China - 37 | 10/10/2021 | 6,000,000 |
| 65 | Sinopharm - China - 38 | 12/10/2021 | 6,000,000 |
| 66 | Sinopharm - China - 39 | 14/10/2021 | 6,000,000 |
| 67 | Sputnik V - Serbia - 14 | 14/10/2021 | 400,000 |
| 68 | AstraZeneca - Poland -1 | 16/10/2021 | 1,000,000 |
| 69 | Sinopharm - China - 40 | 17/10/2021 | 6,000,000 |
| 70 | AstraZeneca - Netherlands (gift from Italy) - 1 | 18/10/2021 | 1,442,000 |
| 71 | Sinopharm - China - 41 | 19/10/2021 | 6,000,000 |
| 72 | Sinopharm - China - 42 | 21/10/2021 | 6,000,000 |
| 73 | Sputnik V - Russia - 15 | 21/10/2021 | 777,600 |
| 74 | Sinopharm - China - 43 | 24/10/2021 | 6,000,000 |
| 75 | Sinopharm - China - 44 | 26/10/2021 | 6,000,000 |
| 76 | AstraZeneca - South Korea - 2 | 27/10/2021 | 1,000,000 |
| 77 | Sinopharm - China - 45 | 28/10/2021 | 6,000,000 |
| 78 | Sputnik V - Russia - 16 | 29/10/2021 | 766,000 |
| 79 | Sinopharm - China - 46 | 31/10/2021 | 6,000,000 |
| 80 | Sinopharm - China - 47 | 02/11/2021 | 148,800 |
| 81 | Sputnik V - Russia - 17 | 04/11/2021 | 777,600 |
| 82 | Sinopharm - China - 48 | 15/11/2021 | 610,422 |
| 83 | Sinopharm - China - 49 | 16/11/2021 | 407,490 |
| 84 | Sinopharm - China - 50 | 16/11/2021 | 593,688 |
| 85 | AstraZeneca - Germany - 1 | 09/12/2021 | 131,000 |
| 86 | AstraZeneca - Netherlands - 2 | 09/12/2021 | 1,087,400 |
| 87 | Sinopharm - China - 51 | 16/11/2021 | 105,600 |
| Cargoes | 87 | Grand Total | 150,108,308 |

**Table S2: Total number of administered doses of COVID-19 vaccines in Iran per vaccine name by 29 May 2022** [[58]](https://paperpile.com/c/gYioBa/7wGi)**.**

| **Vaccine Name** | **Acquisition type** | **Total administered doses** |
| --- | --- | --- |
| **Sputnik V** | Bilateral | 1,380,958 |
| **AstraZeneca** | COVAX + Bilateral | 14,044,541 |
| **Covaxine** | Bilateral | 254,520 |
| **Sinopharm (BBIBP-CorV)** | COVAX + Bilateral + Donation | 120,878,057 |
| **COVIran Barekat** | Local | 9,037,924 |
| **PastoCoVac** | Local | 2,485,211 |
| **SpikoGene** | Local | 1,091,761 |
| **Fakhravac** | Local | 65,200 |
| **Razi CovPars** | Local | 119,676 |
| **Noora** | Local | 10,405 |

**Table S3: Iran’s vaccine roll-out dates per age-group** [[32]](https://paperpile.com/c/gYioBa/qAVn)**.**

| **Age group** | **Roll-out date** | **Days since roll-out of first age group** |
| --- | --- | --- |
| +80 | 2021-05-08 | 0 |
| +75 | 2021-05-18 | 10 |
| +70 | 2021-05-22 | 14 |
| +65 | 2021-07-11 | 64 |
| +60 | 2021-07-14 | 67 |
| +55 | 2021-07-24 | 77 |
| +50 | 2021-07-30 | 83 |
| <50 | 2021-08-03 | 87 |

**Supplementary Figures**


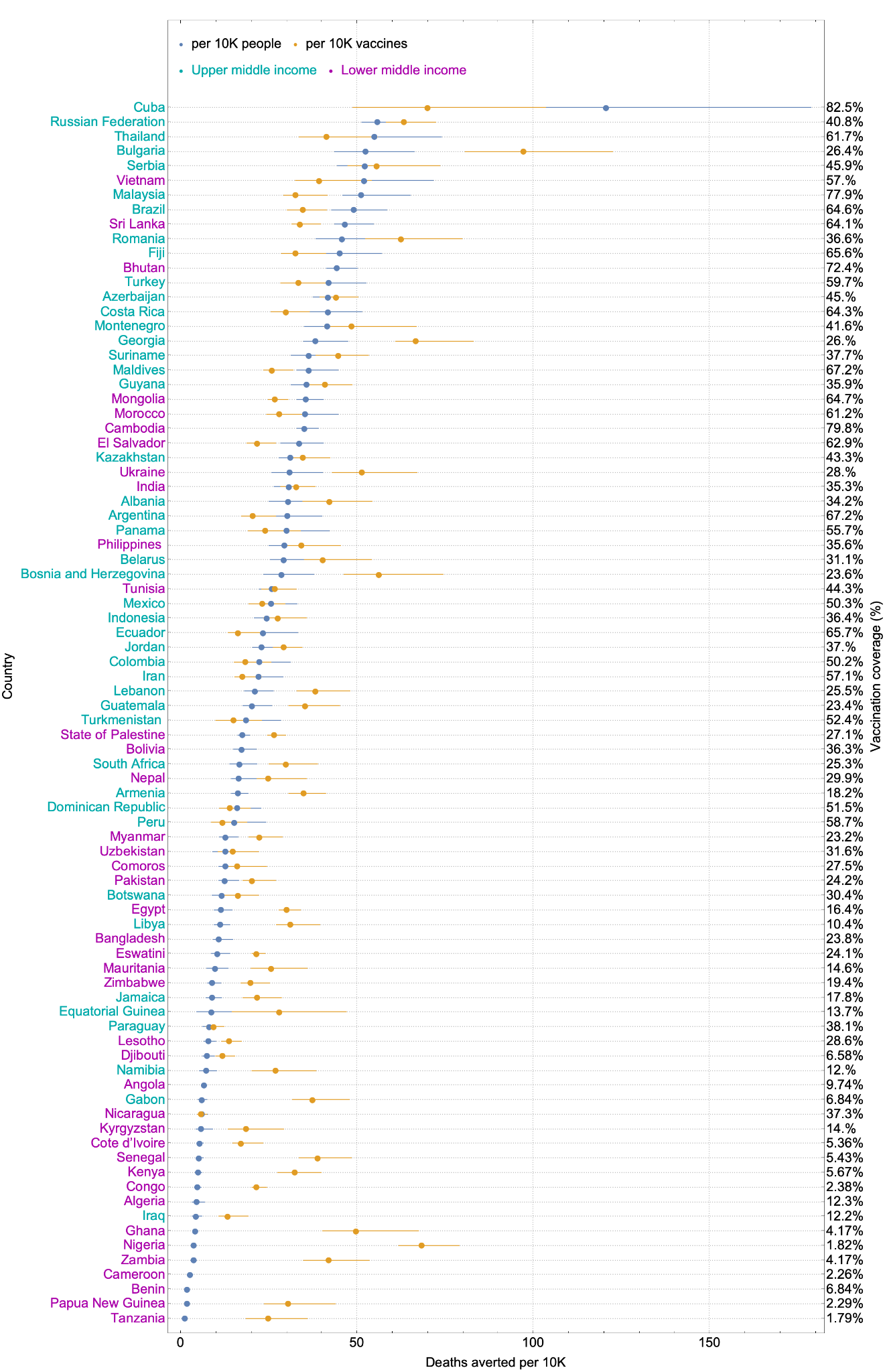


**Figure S1: Deaths averted by vaccination for upper and lower middle income countries, including Iran, up to 2021-12-08.** Data on deaths averted per person and per vaccine are downloaded from <https://github.com/mrc-ide/covid-vaccine-impact-orderly/releases/download/v1.0.1/excess_mortality_summary_table.csv> [[3]](https://paperpile.com/c/gYioBa/RRGZ). The economic status of each country is based on the World Bank income groups in 2020 [[28]](https://paperpile.com/c/gYioBa/M7B5).

A


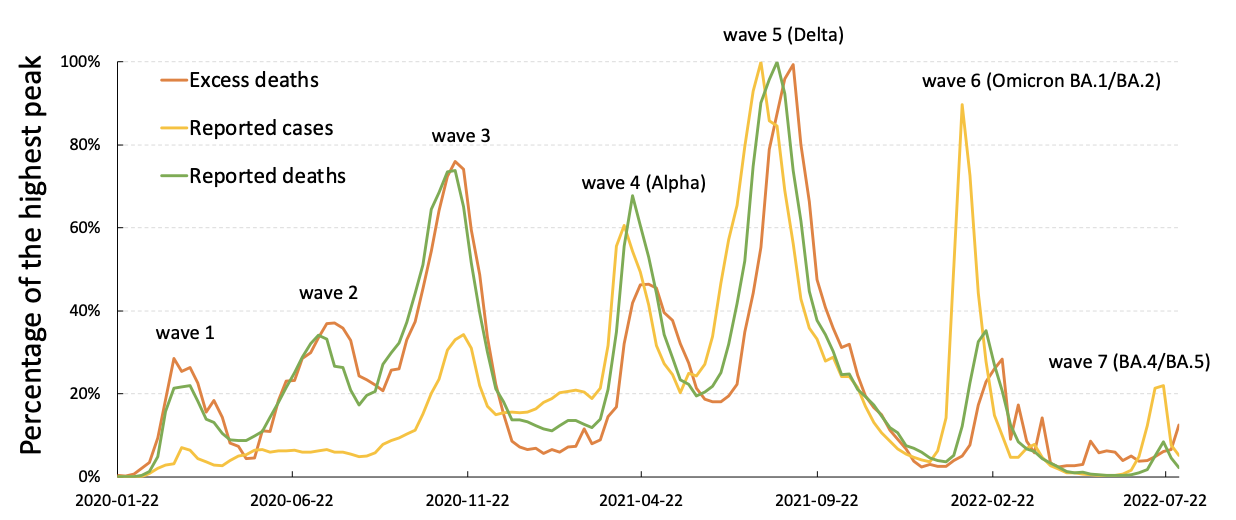


B


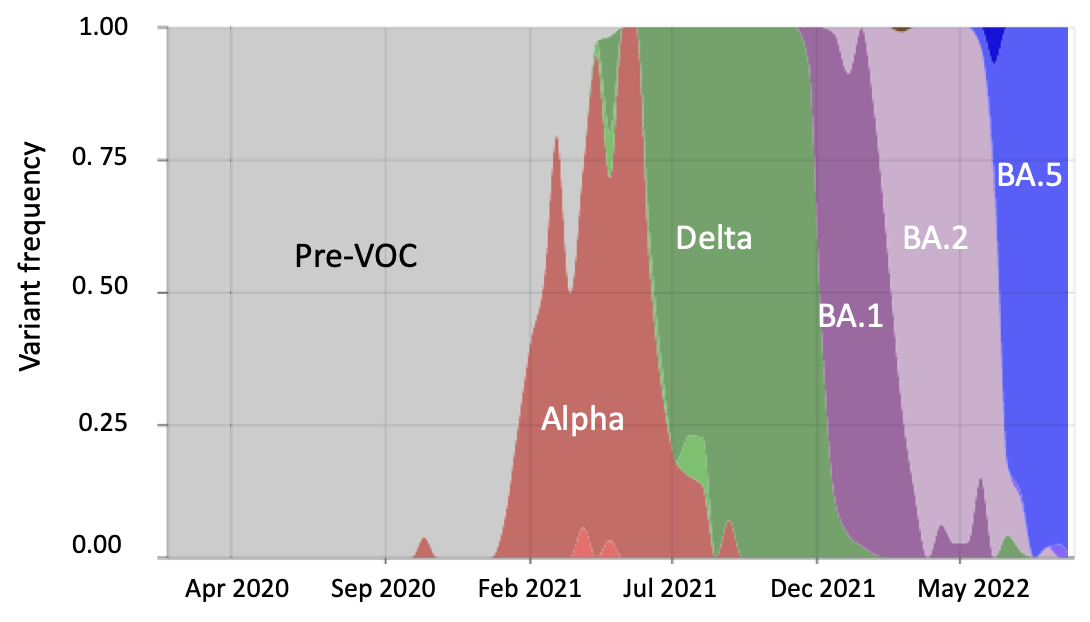


**Figure S2: Association between reported cases, deaths, and excess deaths over time and with respect to variants of SARS-CoV-2.** (A) Reported cases, deaths, and excess deaths (as quantified by the sum of excess deaths per age group) as percentage of highest peak (Delta) in Iran over time. (B) Proportion of the total number of SARS-CoV-2 variants over time in Iran. Data obtained from [[59]](https://paperpile.com/c/gYioBa/QA2s).

Argentina


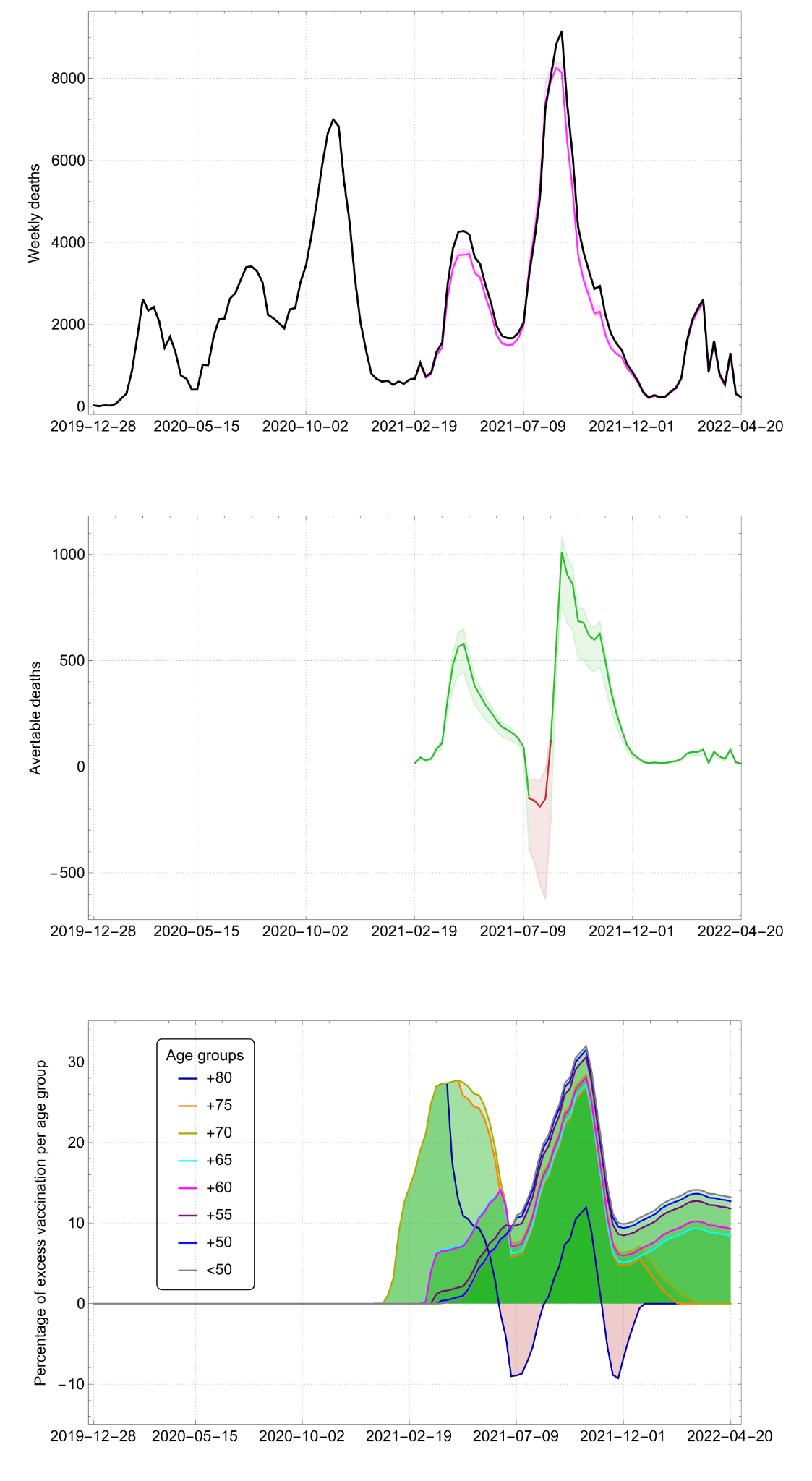


Bahrain


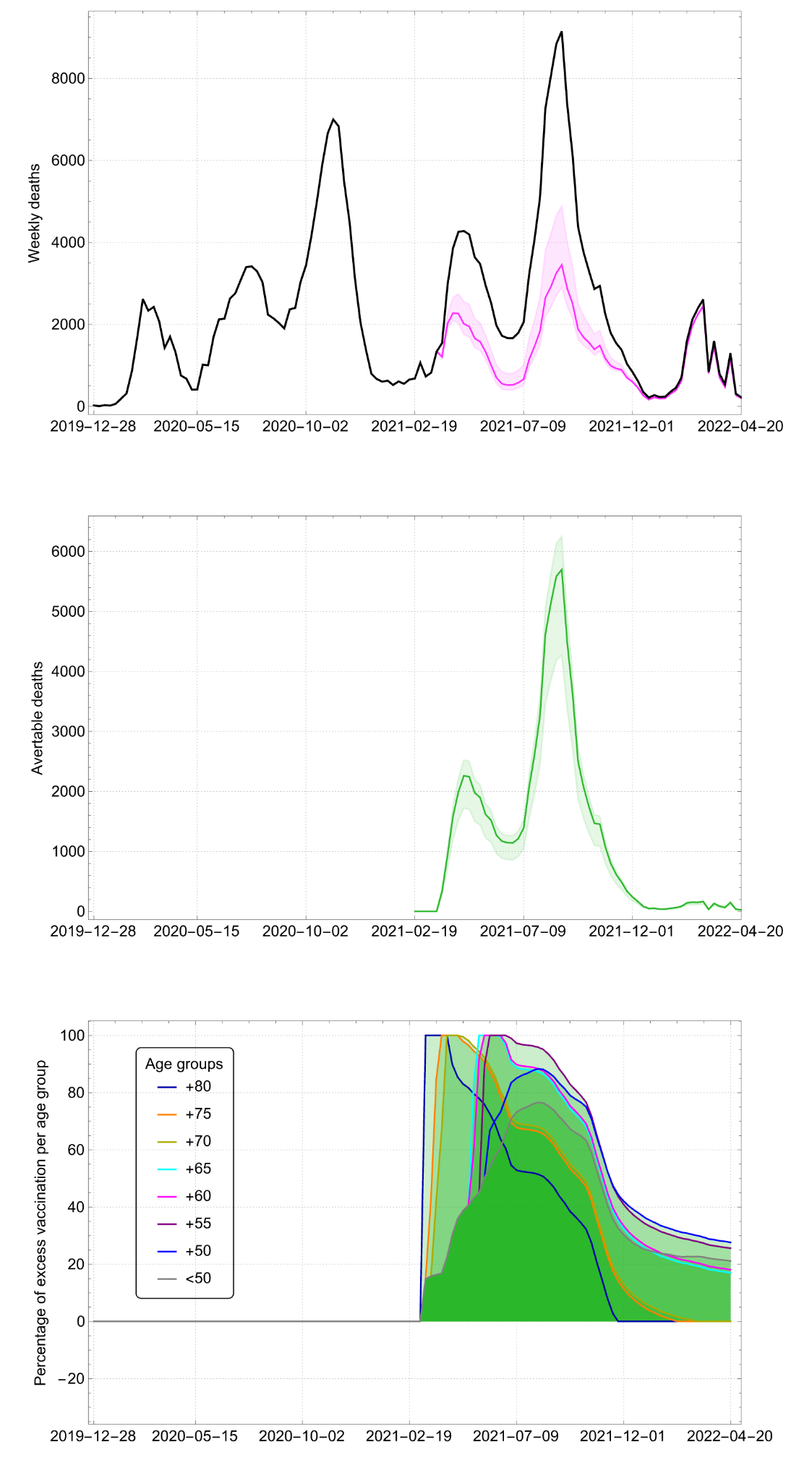


Bangladesh


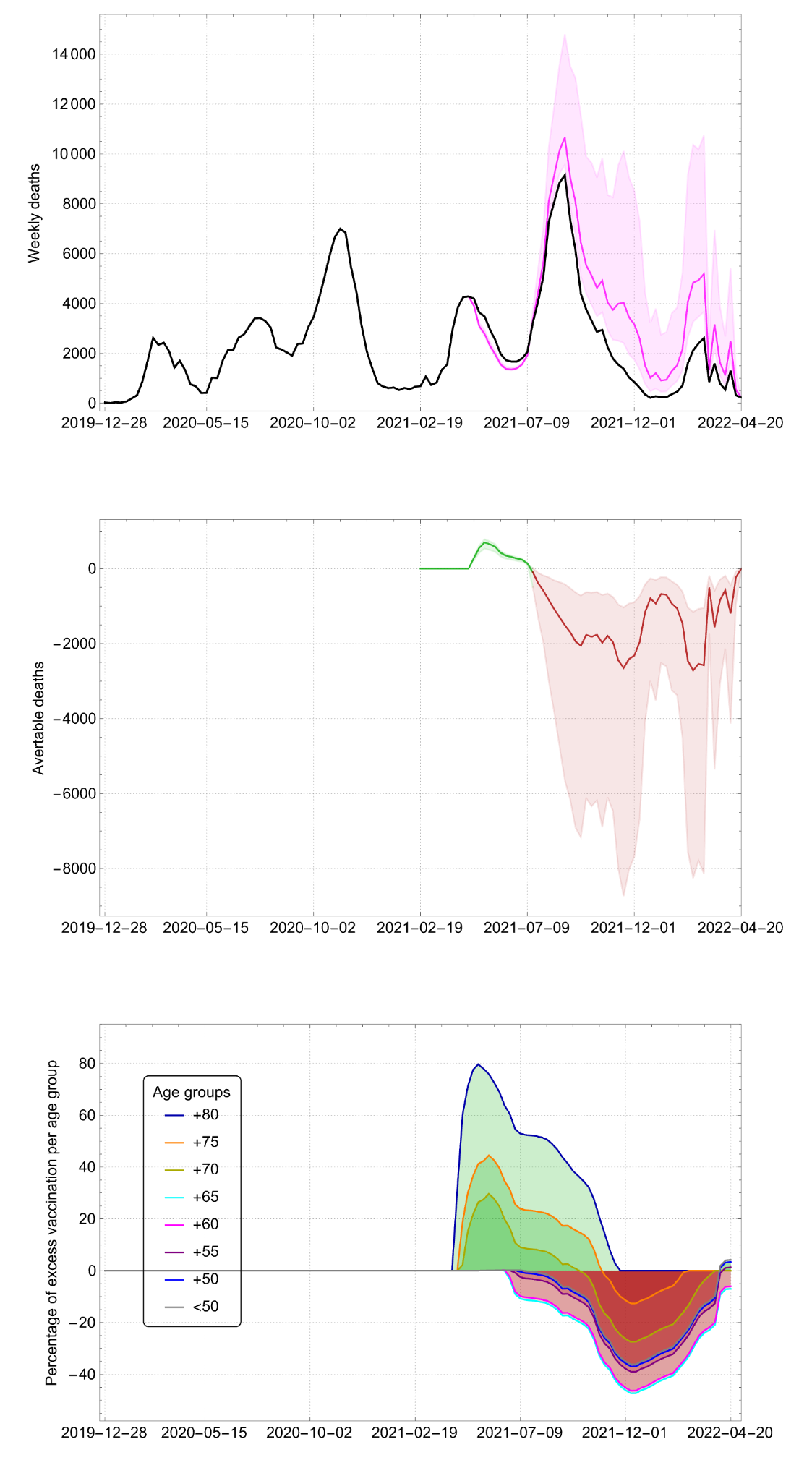


Bolivia


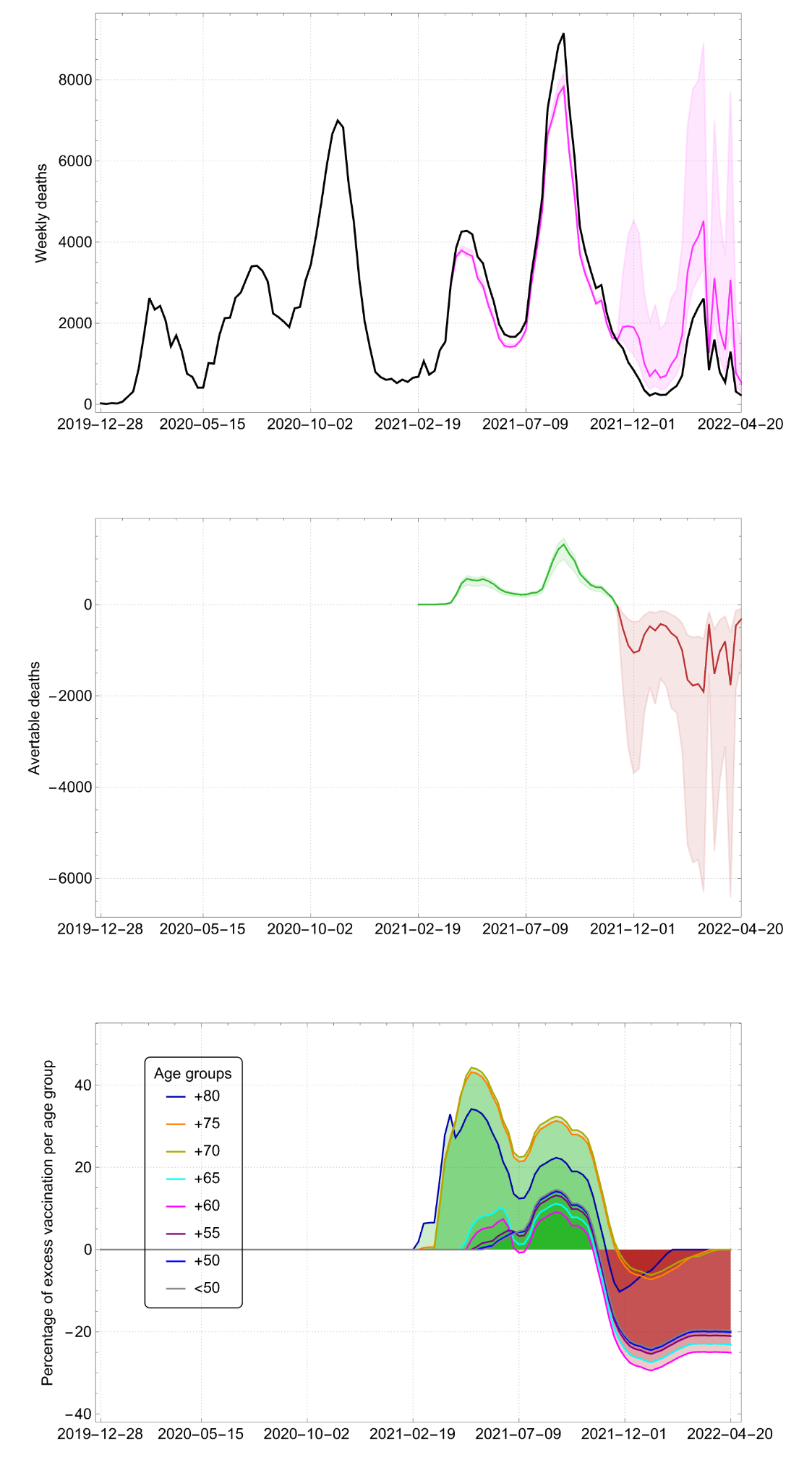


Montenegro


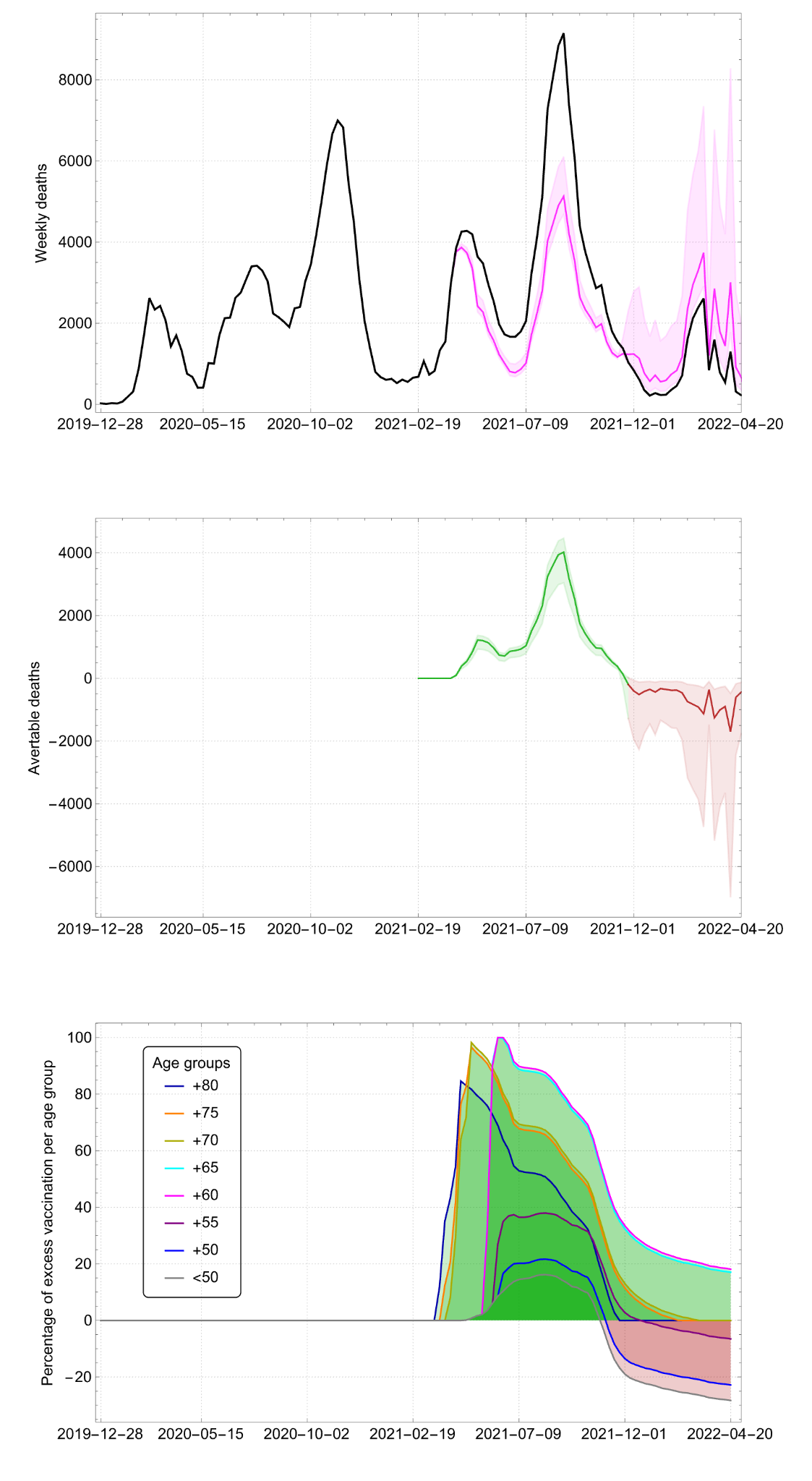


Nepal


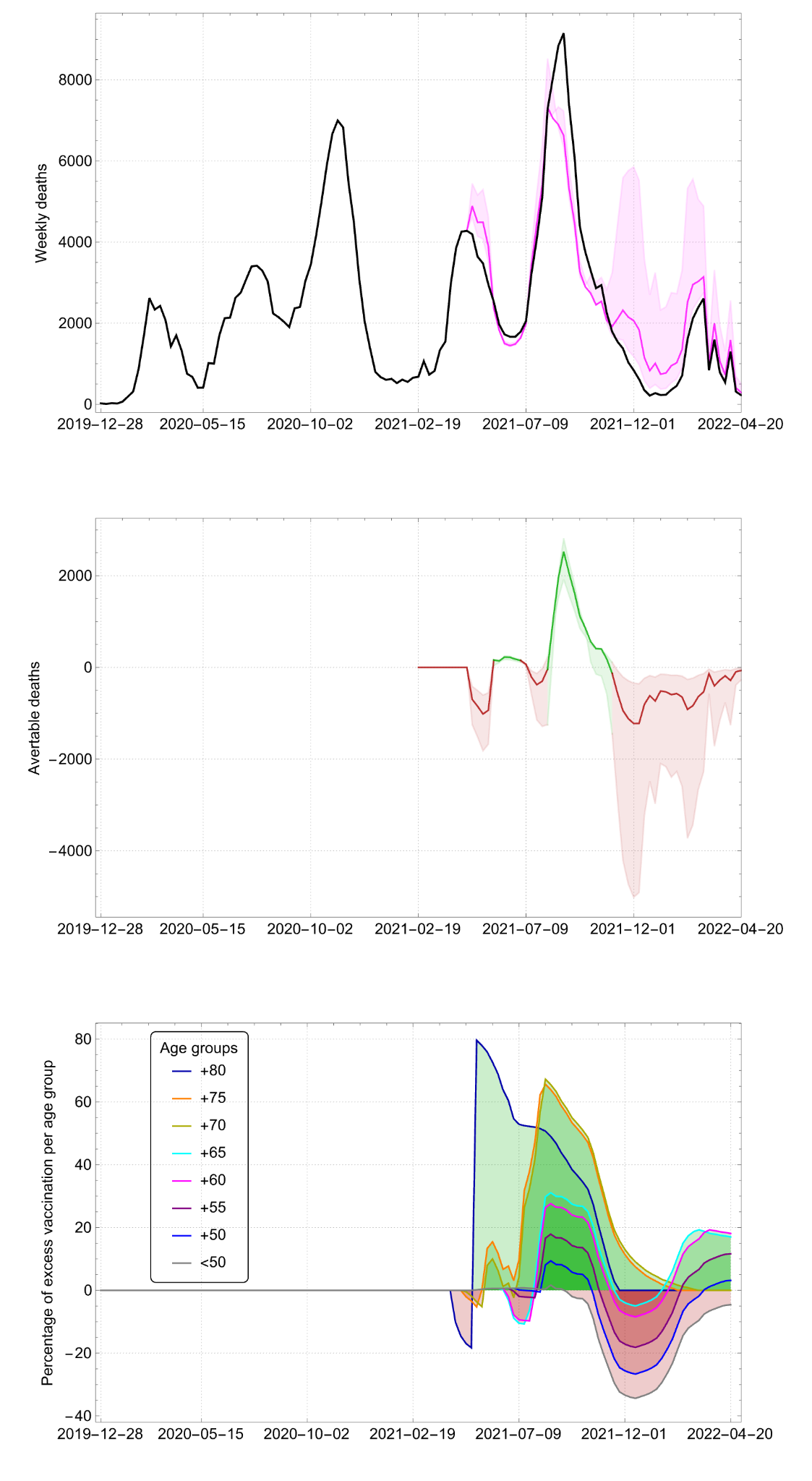


Sri Lanka


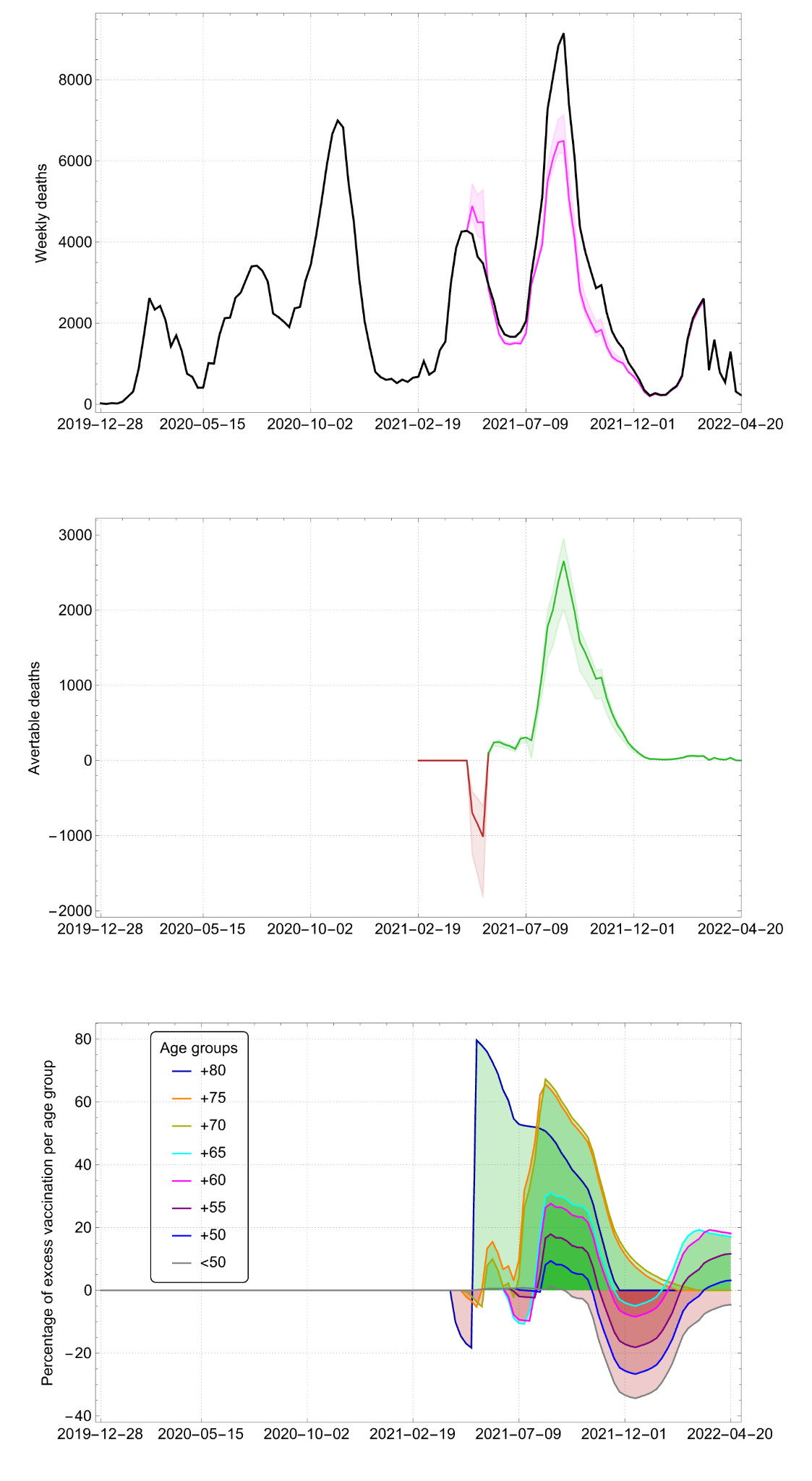


Turkey


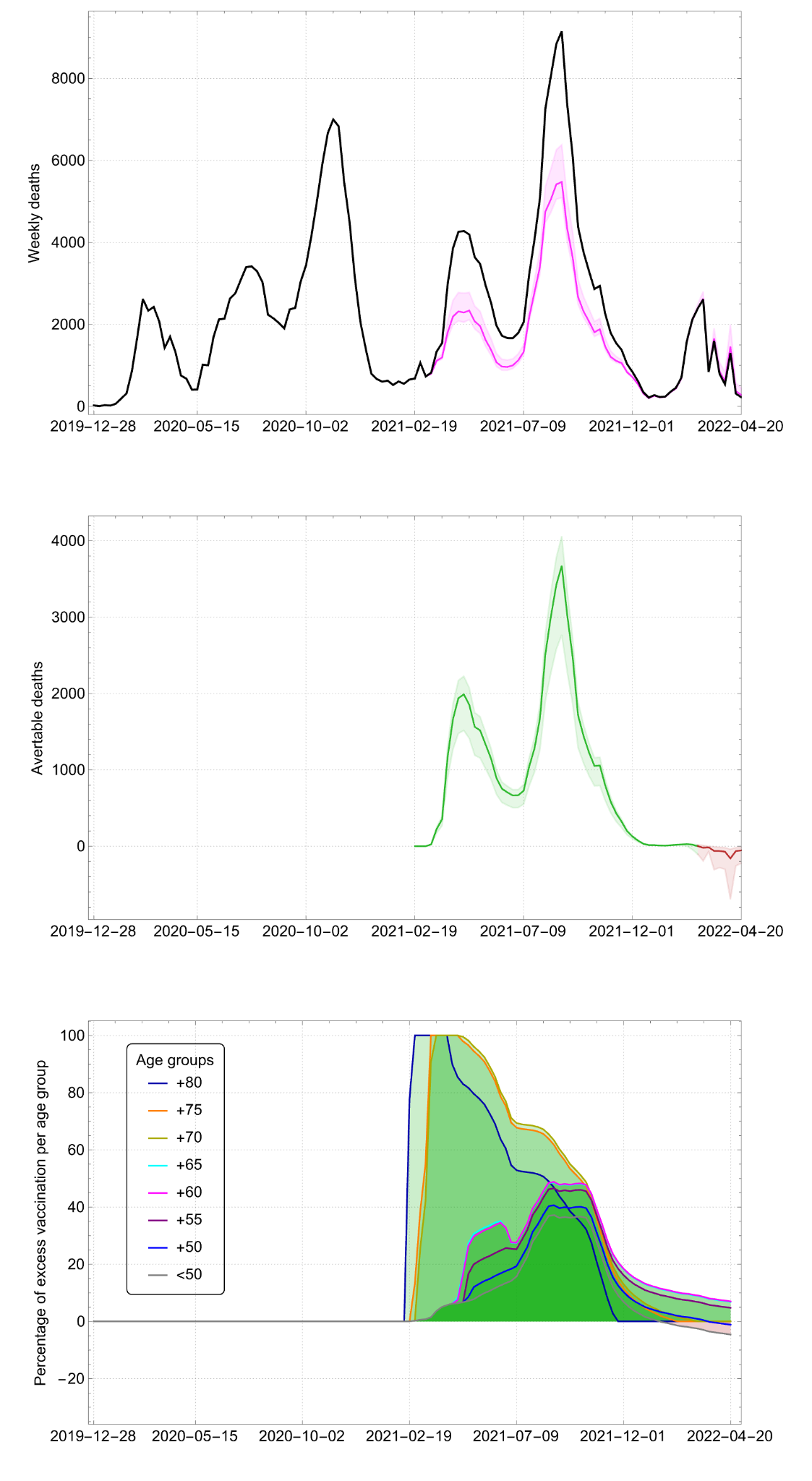


**Figure S3: Avertable COVID-19 deaths in Iran over time based on the per capita vaccine roll-out rates from model countries.** Top panel shows Iran’s weekly excess deaths (black) and counterfactual excess deaths (magenta) had it followed the vaccination rate for each model country. Shaded areas show the 95% confidence interval for the counterfactual excess deaths based on varying degree of vaccine effectiveness against deaths. Central panel shows the avertable deaths based on vaccination rates from a given model country. It shows the difference between weekly excess deaths and counterfactual excess deaths in the top panel. Bottom panel shows the percentage of excess vaccination for a given model country relative to Iran’s vaccination rates per age group. Shaded areas in green (red) show periods where there would have been more (less) vaccination had Iran followed the vaccination rates as the model country.
